# Delayed Presentation of a Retained Fecalith

**DOI:** 10.7759/cureus.15919

**Published:** 2021-06-25

**Authors:** Fawwad A Ansari, Muhammad Ibraiz Bilal, Muhammad Umer Riaz Gondal, Mehwish Latif, Nadeem Iqbal

**Affiliations:** 1 Medicine, Shifa International Hospital, Islamabad, PAK; 2 Gastroenterology, Shifa International Hospital, Islamabad, PAK

**Keywords:** acute appendicitis, appendectomy, fecalith, right iliac fossa pain, complications, colonoscopy

## Abstract

A fecalith is a common cause of acute appendicitis, and laparoscopic surgery is the mainstay of its management. Literature review shows that a fecalith may be retained in the gut following a laparoscopic appendectomy in some rare cases. In most cases, the fecalith becomes symptomatic with time due to the formation of an abscess, fistulous tract, or inflammation of the appendicular stump (stump appendicitis). We report a case of retained appendicular fecalith presenting with symptoms similar to acute appendicitis, 15 years after laparoscopic appendectomy.

## Introduction

A fecalith is a hard stony mass of feces in the intestinal tract. Fecal impaction occurs when a large amount of fecal matter gets compacted and cannot get evacuated spontaneously [1]. In its extreme form, fecal impaction can lead to the formation of a fecalith due to the hardening of fecal material that forms a mass separate from other bowel contents [2].

It can occur in any part of the intestine [1]. Most often, a fecalith arises in the colon (mostly sigmoid) or rectum and very rarely in the small intestine [2].

Here we present a case of a retained appendicular fecalith in a patient who presented with an acute abdomen.

## Case presentation

A 33-year-old male, otherwise healthy, presented to the emergency room with right iliac fossa pain. It was sudden in onset and lasted for about 15 minutes. The pain was severe in intensity and associated with nausea. There was no associated fever, vomiting, or constipation.

The patient had a history of an uneventful appendectomy 15 years back for acute appendicitis. He remained asymptomatic for several years following the procedure. However, four years ago, he presented with ureteric colic, and a CT scan abdomen confirmed a left ureteric stone. The CT scan also incidentally demonstrated a fecalith in the right iliac fossa. He subsequently underwent DJ stenting at that time with complete resolution of symptoms.

During the current visit, abdominal examination showed tenderness in the right iliac fossa with guarding but no abdominal distention, rebound tenderness, or rigidity. A small mass was also palpable in the right lower abdomen. The patient was otherwise vitally stable. 

**Figure 1:**
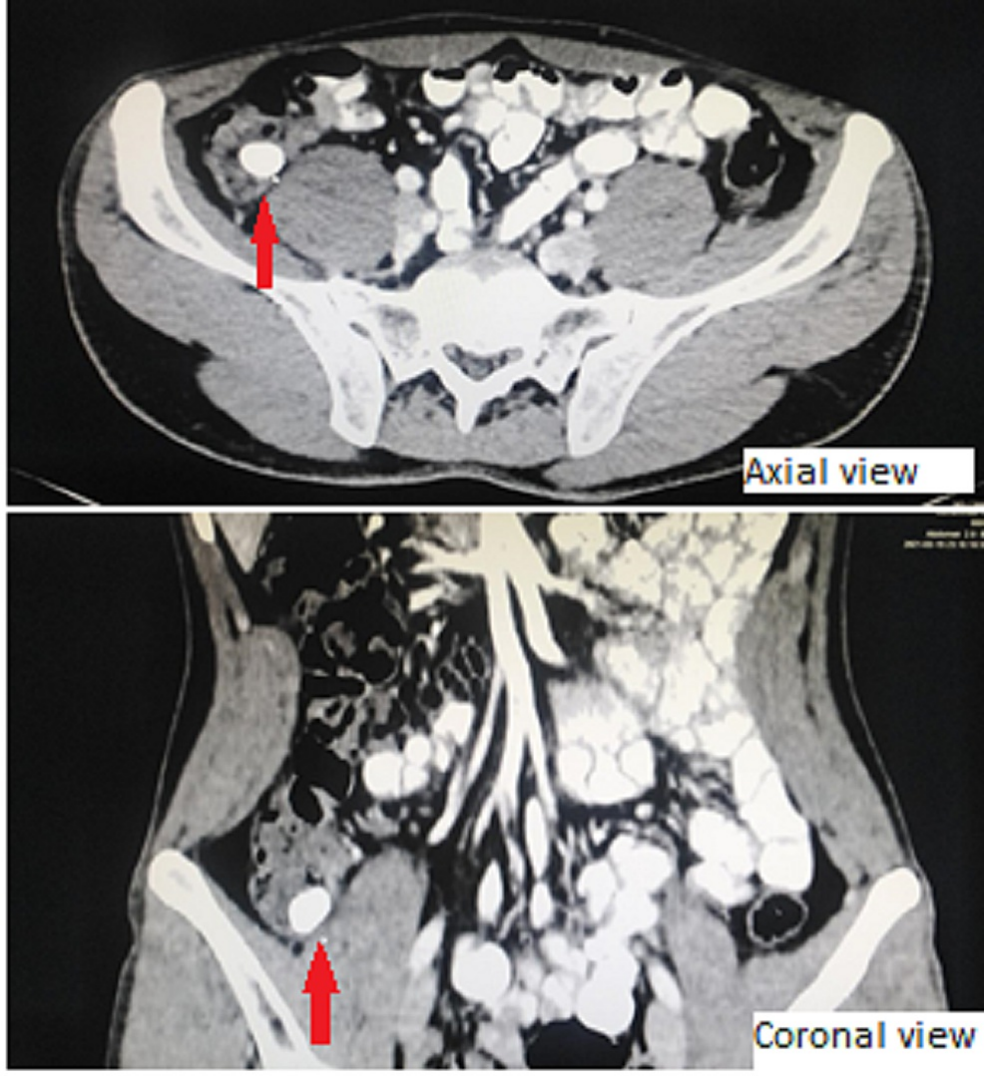
Fecalith as seen on the CT scan

Lab workup showed a total leukocyte count of 10,770/µL and C-reactive peptide levels of 62.7 mg/L. CT scan performed in the emergency room revealed a fecalith in the appendicular stump measuring approximately 17 mm in the maximum transverse diameter (Figure 1). CT scan did not show any inflammatory changes, fat stranding, or fluid collection. The patient got admitted to the hospital for further workup and management.

**Figure 2:**
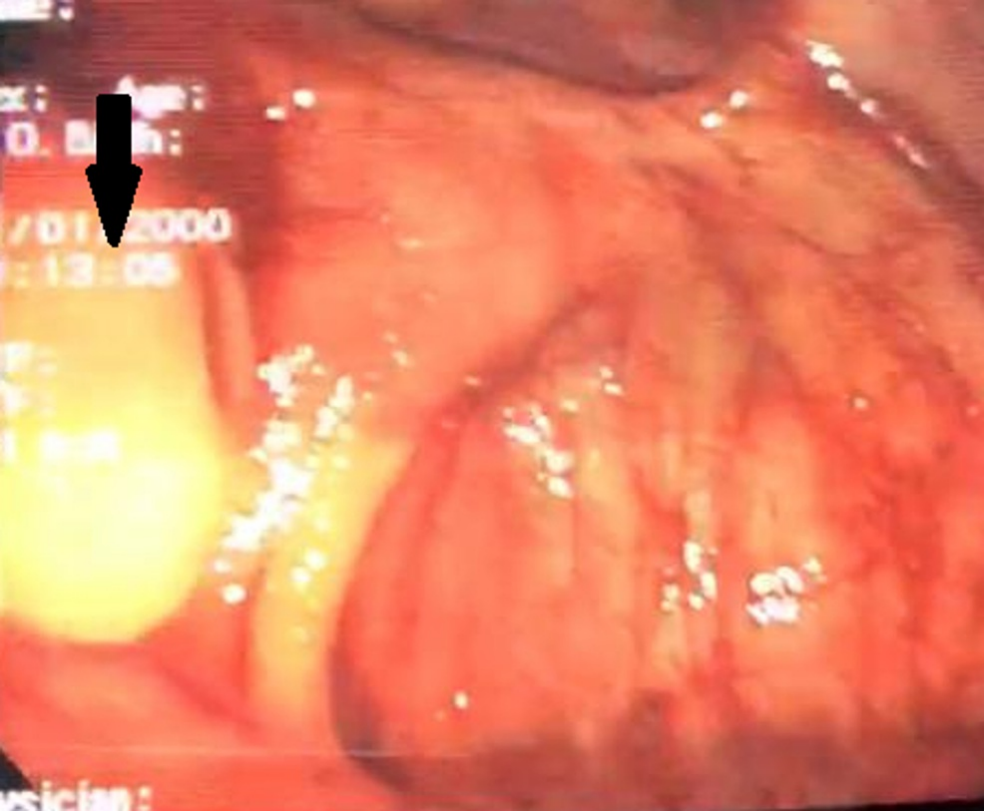
Colonoscopy showing impacted fecalith in the appendicular stump, covered by mucosa and projecting into the cecum

The gastroenterology team was taken on board. Colonoscopy was advised to delineate the exact location of the fecalith and to determine whether it was safe to remove the stone endoscopically. Colonoscopy showed a large impacted and deeply embedded stone in the appendicular stump, covered by normal colonic mucosa and projecting into the cecum (Figure 2).

**Figure 3:**
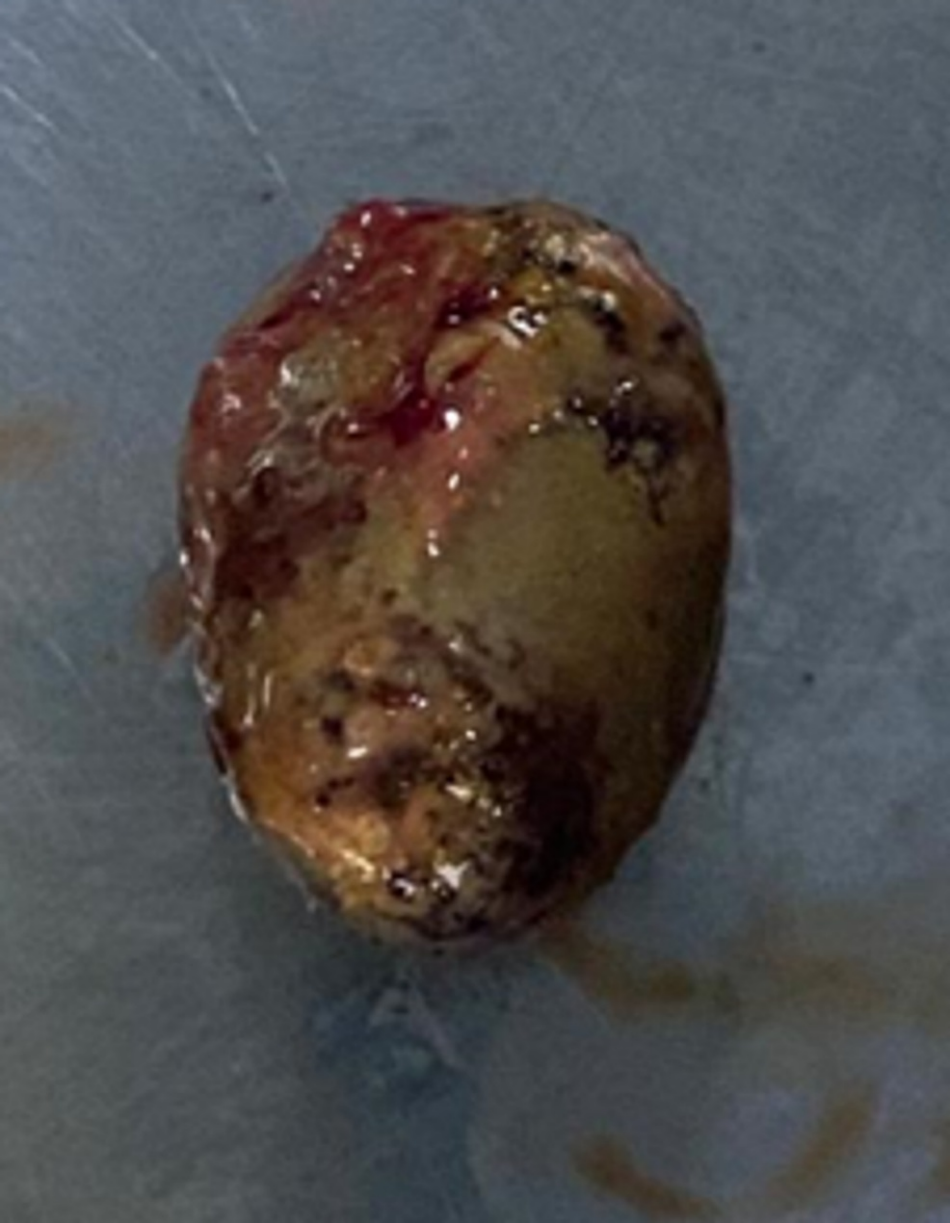
Fecalith retrieved during surgery

The ileocecal valve was normal in appearance, and deep terminal ileal intubation showed normal small bowel mucosa. It was not considered safe to remove the stone endoscopically due to the high risk of cecal perforation. As a result, the patient was referred for surgical removal of the fecalith. The patient underwent laparoscopic surgery with excision of the appendicular stump containing the fecalith (Figure 3). The procedure was uneventful, and the patient remained stable postoperatively. The patient’s symptoms resolved after the surgical excision.

## Discussion

The risk of developing appendicitis during one’s lifetime is around 7%. A fecalith can be found in roughly one-third of the patients that present with symptoms suggestive of appendicitis [3].

Laparoscopic appendectomy is currently the preferred surgical approach for acute appendicitis. A known but a rare complication of laparoscopic appendectomy is a retained fecalith that can occur secondary to rupture of the appendix before surgery or due to failure to remove the fecalith during surgery [4]. 

A retained appendicolith can manifest itself in a multitude of ways. Common presentations include intra-abdominal abscesses, delayed wound healing, fistula formation, appendicular torsion, and stump appendicitis [5,6].

Surgical resection is the mainstay of treatment in the case of symptomatic fecalith or development of its complications [7].

Due to the high risk of infection and potential for stump appendicitis and eventual abscess formation, every effort should be made to remove the appendicoliths at the index operation [8]. Attention should be paid to the appendiceal stump at appendectomy, and any trapped fecalith should be milked out with forceps after appendiceal stump ligation. This practice may help minimize the incidence of postoperative peri-appendiceal abscess and stump appendicitis [5].

The lengthiest interval we could find in the literature that reports abscess formation due to a retained fecalith was nine years after appendectomy [9]. Usually, the time between appendectomy and the manifestations of retained appendicolith varies from 10 days to several years [5].

Keeping the current literature in view, our case seems to be peculiar. Our patient presented with symptoms attributable to fecalith 15 years following an appendectomy. It is interesting to note that the fecalith remained silent over a considerable number of years. We are not sure of the reason for the patient’s current symptoms as there was no evidence of inflammation of the appendicular stump or any pus collection on radiological imaging. However, due to the intensity of the pain, right iliac fossa tenderness, and raised inflammatory marker, some degree of inflammation was considered likely, and it was decided that we remove the fecalith. There is also a possibility that the episode of acute pain was due to mechanical distension of the stump. 

We attempted to retrieve the stone endoscopically, but due to the risk of bowel perforation, laparoscopic surgery was done for appendicolith removal. There was a prompt resolution of symptoms after the patient’s surgery, indicating that the fecalith was the source of the problem. 

Regardless of the cause, any patient with a prior scar from appendectomy with right iliac fossa pain should be suspected for a retained fecalith or one of its complications. These rare complications of surgery can easily be missed, and one must be aware of the possibility of such a rare diagnosis.

## Conclusions

Retained fecalith after appendix removal can present variably, both with respect to the onset and symptoms of presentation. Despite the rarity of this phenomenon, it is worthwhile to consider a retained fecalith as a possible differential diagnosis in any patient who presents with right iliac fossa pain with prior history of appendectomy, alongside the more common pathologies. Our case report provides a reason to consider this cause of acute abdomen even in a patient with a considerably old history of surgical appendix removal.
